# Supplementary material for: Dynalign II: common secondary structure prediction for RNA homologs with domain insertions
Source: Nucleic Acids Res. 2014 Nov 21;42(22):13939–48. doi: 10.1093/nar/gku1172 (PMC4267632; doi:10.1093/nar/gku1172)
Supplement: SUPPLEMENTARY DATA [file supp_gku1172_nar-02021-z-2014-File012.zip › manual/javadoc/RNAstructure_java_utilities/NumberField.DoubleField.html]

NumberField.DoubleField


JavaScript is disabled on your browser.


- Overview
- Package
- Class
- Tree
- Index
- Help

- Prev Class
- Next Class

- Frames
- No Frames

- All Classes

- Summary:
- Nested |
- Field |
- Constr |
- Method

- Detail:
- Field |
- Constr |
- Method


RNAstructure\_java\_utilities

## Class NumberField.DoubleField

- java.lang.Object
- - java.awt.Component
  - - java.awt.Container
    - - javax.swing.JComponent
      - - javax.swing.text.JTextComponent
        - - javax.swing.JTextField
          - - RNAstructure\_java\_utilities.NumberField
            - - RNAstructure\_java\_utilities.NumberField.DoubleField

- All Implemented Interfaces:
  :   ImageObserver, MenuContainer, Serializable, Accessible, Scrollable, SwingConstants

  Enclosing class:
  :   NumberField

  ---

    

  ```
  public static class NumberField.DoubleField
  extends NumberField
  ```

  An inner class that handles a field which contains a double value.

  See Also:
  :   Serialized Form

- - ### Nested Class Summary

    - ### Nested classes/interfaces inherited from class RNAstructure\_java\_utilities.NumberField

      `NumberField.DoubleField, NumberField.FloatField, NumberField.IntegerField`
    - ### Nested classes/interfaces inherited from class javax.swing.JTextField

      `JTextField.AccessibleJTextField`
    - ### Nested classes/interfaces inherited from class javax.swing.text.JTextComponent

      `JTextComponent.AccessibleJTextComponent, JTextComponent.DropLocation, JTextComponent.KeyBinding`
    - ### Nested classes/interfaces inherited from class javax.swing.JComponent

      `JComponent.AccessibleJComponent`
    - ### Nested classes/interfaces inherited from class java.awt.Container

      `Container.AccessibleAWTContainer`
    - ### Nested classes/interfaces inherited from class java.awt.Component

      `Component.AccessibleAWTComponent, Component.BaselineResizeBehavior, Component.BltBufferStrategy, Component.FlipBufferStrategy`
  - ### Field Summary

    Fields

    | Modifier and Type | Field and Description |
    | --- | --- |
    | `private static long` | `serialVersionUID` |

    - ### Fields inherited from class RNAstructure\_java\_utilities.NumberField

      `initial, maximum, minimum`
    - ### Fields inherited from class javax.swing.JTextField

      `notifyAction`
    - ### Fields inherited from class javax.swing.text.JTextComponent

      `DEFAULT_KEYMAP, FOCUS_ACCELERATOR_KEY`
    - ### Fields inherited from class javax.swing.JComponent

      `accessibleContext, listenerList, TOOL_TIP_TEXT_KEY, ui, UNDEFINED_CONDITION, WHEN_ANCESTOR_OF_FOCUSED_COMPONENT, WHEN_FOCUSED, WHEN_IN_FOCUSED_WINDOW`
    - ### Fields inherited from class java.awt.Component

      `BOTTOM_ALIGNMENT, CENTER_ALIGNMENT, LEFT_ALIGNMENT, RIGHT_ALIGNMENT, TOP_ALIGNMENT`
    - ### Fields inherited from interface javax.swing.SwingConstants

      `BOTTOM, CENTER, EAST, HORIZONTAL, LEADING, LEFT, NEXT, NORTH, NORTH_EAST, NORTH_WEST, PREVIOUS, RIGHT, SOUTH, SOUTH_EAST, SOUTH_WEST, TOP, TRAILING, VERTICAL, WEST`
    - ### Fields inherited from interface java.awt.image.ImageObserver

      `ABORT, ALLBITS, ERROR, FRAMEBITS, HEIGHT, PROPERTIES, SOMEBITS, WIDTH`
  - ### Constructor Summary

    Constructors

    Constructor and Description

    `NumberField.DoubleField(String text,
    Number value)`

    Unbounded Constructor.

    `NumberField.DoubleField(String text,
    Number value,
    Number min)`

    Minimum Bounds Constructor.

    `NumberField.DoubleField(String text,
    Number value,
    Number min,
    Number max)`

    Minimum and Maximum Bounds Constructor.
  - ### Method Summary

    Methods

    | Modifier and Type | Method and Description |
    | --- | --- |
    | `protected boolean` | `checkValue()` Check the value of a field to make sure it's valid. |
    | `protected boolean` | `doAdditionalCheck(char key)` Do any additional necessary checks on a key character to make sure it's valid to be output. |
    | `Double` | `getValue()` Get the text field's numeric value. |

    - ### Methods inherited from class RNAstructure\_java\_utilities.NumberField

      `resetField, resetField`
    - ### Methods inherited from class javax.swing.JTextField

      `actionPropertyChanged, addActionListener, configurePropertiesFromAction, createActionPropertyChangeListener, createDefaultModel, fireActionPerformed, getAccessibleContext, getAction, getActionListeners, getActions, getColumns, getColumnWidth, getHorizontalAlignment, getHorizontalVisibility, getPreferredSize, getScrollOffset, getUIClassID, isValidateRoot, paramString, postActionEvent, removeActionListener, scrollRectToVisible, setAction, setActionCommand, setColumns, setDocument, setFont, setHorizontalAlignment, setScrollOffset`
    - ### Methods inherited from class javax.swing.text.JTextComponent

      `addCaretListener, addInputMethodListener, addKeymap, copy, cut, fireCaretUpdate, getCaret, getCaretColor, getCaretListeners, getCaretPosition, getDisabledTextColor, getDocument, getDragEnabled, getDropLocation, getDropMode, getFocusAccelerator, getHighlighter, getInputMethodRequests, getKeymap, getKeymap, getMargin, getNavigationFilter, getPreferredScrollableViewportSize, getPrintable, getScrollableBlockIncrement, getScrollableTracksViewportHeight, getScrollableTracksViewportWidth, getScrollableUnitIncrement, getSelectedText, getSelectedTextColor, getSelectionColor, getSelectionEnd, getSelectionStart, getText, getText, getToolTipText, getUI, isEditable, loadKeymap, modelToView, moveCaretPosition, paste, print, print, print, processInputMethodEvent, read, removeCaretListener, removeKeymap, removeNotify, replaceSelection, restoreComposedText, saveComposedText, select, selectAll, setCaret, setCaretColor, setCaretPosition, setComponentOrientation, setDisabledTextColor, setDragEnabled, setDropMode, setEditable, setFocusAccelerator, setHighlighter, setKeymap, setMargin, setNavigationFilter, setSelectedTextColor, setSelectionColor, setSelectionEnd, setSelectionStart, setText, setUI, updateUI, viewToModel, write`
    - ### Methods inherited from class javax.swing.JComponent

      `addAncestorListener, addNotify, addVetoableChangeListener, computeVisibleRect, contains, createToolTip, disable, enable, firePropertyChange, firePropertyChange, firePropertyChange, fireVetoableChange, getActionForKeyStroke, getActionMap, getAlignmentX, getAlignmentY, getAncestorListeners, getAutoscrolls, getBaseline, getBaselineResizeBehavior, getBorder, getBounds, getClientProperty, getComponentGraphics, getComponentPopupMenu, getConditionForKeyStroke, getDebugGraphicsOptions, getDefaultLocale, getFontMetrics, getGraphics, getHeight, getInheritsPopupMenu, getInputMap, getInputMap, getInputVerifier, getInsets, getInsets, getListeners, getLocation, getMaximumSize, getMinimumSize, getNextFocusableComponent, getPopupLocation, getRegisteredKeyStrokes, getRootPane, getSize, getToolTipLocation, getToolTipText, getTopLevelAncestor, getTransferHandler, getVerifyInputWhenFocusTarget, getVetoableChangeListeners, getVisibleRect, getWidth, getX, getY, grabFocus, isDoubleBuffered, isLightweightComponent, isManagingFocus, isOpaque, isOptimizedDrawingEnabled, isPaintingForPrint, isPaintingOrigin, isPaintingTile, isRequestFocusEnabled, paint, paintBorder, paintChildren, paintComponent, paintImmediately, paintImmediately, print, printAll, printBorder, printChildren, printComponent, processComponentKeyEvent, processKeyBinding, processKeyEvent, processMouseEvent, processMouseMotionEvent, putClientProperty, registerKeyboardAction, registerKeyboardAction, removeAncestorListener, removeVetoableChangeListener, repaint, repaint, requestDefaultFocus, requestFocus, requestFocus, requestFocusInWindow, requestFocusInWindow, resetKeyboardActions, reshape, revalidate, setActionMap, setAlignmentX, setAlignmentY, setAutoscrolls, setBackground, setBorder, setComponentPopupMenu, setDebugGraphicsOptions, setDefaultLocale, setDoubleBuffered, setEnabled, setFocusTraversalKeys, setForeground, setInheritsPopupMenu, setInputMap, setInputVerifier, setMaximumSize, setMinimumSize, setNextFocusableComponent, setOpaque, setPreferredSize, setRequestFocusEnabled, setToolTipText, setTransferHandler, setUI, setVerifyInputWhenFocusTarget, setVisible, unregisterKeyboardAction, update`
    - ### Methods inherited from class java.awt.Container

      `add, add, add, add, add, addContainerListener, addImpl, addPropertyChangeListener, addPropertyChangeListener, applyComponentOrientation, areFocusTraversalKeysSet, countComponents, deliverEvent, doLayout, findComponentAt, findComponentAt, getComponent, getComponentAt, getComponentAt, getComponentCount, getComponents, getComponentZOrder, getContainerListeners, getFocusTraversalKeys, getFocusTraversalPolicy, getLayout, getMousePosition, insets, invalidate, isAncestorOf, isFocusCycleRoot, isFocusCycleRoot, isFocusTraversalPolicyProvider, isFocusTraversalPolicySet, layout, list, list, locate, minimumSize, paintComponents, preferredSize, printComponents, processContainerEvent, processEvent, remove, remove, removeAll, removeContainerListener, setComponentZOrder, setFocusCycleRoot, setFocusTraversalPolicy, setFocusTraversalPolicyProvider, setLayout, transferFocusDownCycle, validate, validateTree`
    - ### Methods inherited from class java.awt.Component

      `action, add, addComponentListener, addFocusListener, addHierarchyBoundsListener, addHierarchyListener, addKeyListener, addMouseListener, addMouseMotionListener, addMouseWheelListener, bounds, checkImage, checkImage, coalesceEvents, contains, createImage, createImage, createVolatileImage, createVolatileImage, disableEvents, dispatchEvent, enable, enableEvents, enableInputMethods, firePropertyChange, firePropertyChange, firePropertyChange, firePropertyChange, firePropertyChange, firePropertyChange, getBackground, getBounds, getColorModel, getComponentListeners, getComponentOrientation, getCursor, getDropTarget, getFocusCycleRootAncestor, getFocusListeners, getFocusTraversalKeysEnabled, getFont, getForeground, getGraphicsConfiguration, getHierarchyBoundsListeners, getHierarchyListeners, getIgnoreRepaint, getInputContext, getInputMethodListeners, getKeyListeners, getLocale, getLocation, getLocationOnScreen, getMouseListeners, getMouseMotionListeners, getMousePosition, getMouseWheelListeners, getName, getParent, getPeer, getPropertyChangeListeners, getPropertyChangeListeners, getSize, getToolkit, getTreeLock, gotFocus, handleEvent, hasFocus, hide, imageUpdate, inside, isBackgroundSet, isCursorSet, isDisplayable, isEnabled, isFocusable, isFocusOwner, isFocusTraversable, isFontSet, isForegroundSet, isLightweight, isMaximumSizeSet, isMinimumSizeSet, isPreferredSizeSet, isShowing, isValid, isVisible, keyDown, keyUp, list, list, list, location, lostFocus, mouseDown, mouseDrag, mouseEnter, mouseExit, mouseMove, mouseUp, move, nextFocus, paintAll, postEvent, prepareImage, prepareImage, processComponentEvent, processFocusEvent, processHierarchyBoundsEvent, processHierarchyEvent, processMouseWheelEvent, remove, removeComponentListener, removeFocusListener, removeHierarchyBoundsListener, removeHierarchyListener, removeInputMethodListener, removeKeyListener, removeMouseListener, removeMouseMotionListener, removeMouseWheelListener, removePropertyChangeListener, removePropertyChangeListener, repaint, repaint, repaint, resize, resize, setBounds, setBounds, setCursor, setDropTarget, setFocusable, setFocusTraversalKeysEnabled, setIgnoreRepaint, setLocale, setLocation, setLocation, setName, setSize, setSize, show, show, size, toString, transferFocus, transferFocusBackward, transferFocusUpCycle`
    - ### Methods inherited from class java.lang.Object

      `clone, equals, finalize, getClass, hashCode, notify, notifyAll, wait, wait, wait`

- - ### Field Detail


    - #### serialVersionUID

      ```
      private static final long serialVersionUID
      ```

      See Also:
      :   Constant Field Values
  - ### Constructor Detail


    - #### NumberField.DoubleField

      ```
      public NumberField.DoubleField(String text,
                             Number value)
      ```

      Unbounded Constructor.

      Parameters:
      :   `text` - The name of the field.
      :   `value` - The initial numeric value of the field.


    - #### NumberField.DoubleField

      ```
      public NumberField.DoubleField(String text,
                             Number value,
                             Number min)
      ```

      Minimum Bounds Constructor.

      Parameters:
      :   `text` - The name of the field.
      :   `value` - The initial numeric value of the field.
      :   `min` - The minimum value of the field.


    - #### NumberField.DoubleField

      ```
      public NumberField.DoubleField(String text,
                             Number value,
                             Number min,
                             Number max)
      ```

      Minimum and Maximum Bounds Constructor.

      Parameters:
      :   `text` - The name of the field.
      :   `value` - The initial numeric value of the field.
      :   `min` - The minimum value of the field.
      :   `max` - The maximum value of the field.
  - ### Method Detail


    - #### checkValue

      ```
      protected boolean checkValue()
      ```

      **Description copied from class: `NumberField`**

      Check the value of a field to make sure it's valid.

      **Specified by:**
      :   `checkValue` in class `NumberField`

      Returns:
      :   True if the field value is valid, false if not.


    - #### doAdditionalCheck

      ```
      protected boolean doAdditionalCheck(char key)
      ```

      **Description copied from class: `NumberField`**

      Do any additional necessary checks on a key character to make sure it's
      valid to be output.

      **Overrides:**
      :   `doAdditionalCheck` in class `NumberField`

      Parameters:
      :   `key` - The key character.


    - #### getValue

      ```
      public Double getValue()
      ```

      **Description copied from class: `NumberField`**

      Get the text field's numeric value.

      **Specified by:**
      :   `getValue` in class `NumberField`

      Returns:
      :   The numeric value.


- Overview
- Package
- Class
- Tree
- Index
- Help

- Prev Class
- Next Class

- Frames
- No Frames

- All Classes

- Summary:
- Nested |
- Field |
- Constr |
- Method

- Detail:
- Field |
- Constr |
- Method
